# The Knowledge and Attitude of Undergraduate Dental Students toward Dental Ergonomic Principles in Occupational Health

**DOI:** 10.3390/healthcare12161566

**Published:** 2024-08-07

**Authors:** Monika Tysiąc-Miśta, Maja Kruplewicz, Aleksandra Grzyb, Arkadiusz Dziedzic, Marta Tanasiewicz

**Affiliations:** 1 Department of Conservative Dentistry with Endodontics, Medical University of Silesia, 40-055 Katowice, Poland; mtysiac-mista@sum.edu.pl (M.T.-M.); martatanasiewicz@sum.edu.pl (M.T.); 2 Students’ Scientific Group, Department of Conservative Dentistry with Endodontics, Medical University of Silesia, 40-055 Katowice, Poland

**Keywords:** dentistry, ergonomics, dental students, occupational health, musculoskeletal disorders, work-related diseases, occupational health hazards

## Abstract

Background: Undergraduate dental curricula and courses containing ergonomic principles are introduced to students from the very beginning of dental education. Still, dentists present a high prevalence of musculoskeletal disorders, which are a direct burden on quality of life, leading to early retirement from the profession. This study aimed to assess the state of students’ knowledge and awareness regarding the role of ergonomics in dentistry and its practical implementation. Methods: a cross-sectional study using a predesigned self-administered questionnaire was conducted among fourth- and fifth-year dental students of the Medical University of Silesia in Katowice, Poland (response rate of 69.2%). Results: A total of 94.6% of students declared a good, fair, or moderate level of knowledge of the subject, with a significant difference in favor of the fifth-year students (*p* = 0.008). Moreover, 76.1% of respondents showed a good or very good attitude toward ergonomics as a university curriculum subject. A total of 72.3% of respondents declared that the practical application of ergonomic rules in clinical dentistry is very important (five in a five-point scale). Women found dental ergonomics more important than men (*p* < 0.001). However, 79.3% of participants admitted not following basic ergonomic principles during clinical procedures. Conclusion: The research revealed a discrepancy between theoretical knowledge and awareness and the practical application of ergonomics in a clinical environment. Only by inculcating correct occupational standards and abiding by them from the commencement of dental education can consistency between theory and practice be achieved. This will hopefully ensure the health and well-being of dental team members throughout their professional lives.

## 1. Introduction

Ergonomics is an interdisciplinary science concerned with designing equipment and techniques for maximum efficiency and safety to improve human well-being and overall system performance in the work area [1]. Ergonomics in dentistry is defined as a reduction in cognitive and physical stress, preventing occupational diseases, and thereby improving efficiency, with better quality and greater comfort of the work-related environment for the benefit of both practitioners and patients [2,3]. The role of early education on ergonomics is to inculcate good habits, which will ensure the health and well-being of dental staff members in the following decades of their professional lives [4].

Dentistry is a highly demanding profession from both physical and mental standpoints [5,6]. Dentists, among all healthcare professionals, present a very high prevalence of musculoskeletal disorders (MSDs), such as lower back (>60%), shoulder (>55.1%), and wrist (>39.1%) pain [7]. The main risk factors of work-related musculoskeletal disorders (WMSDs) are repeated over-extension and overuse of certain groups of muscles, huge physical effort to perform precise and time-consuming procedures in a small operating field, asymmetric position outside the neutral range of movement, and organizational factors, such as a variety of cases, lack of total control, workload, time pressure, and financial constraints [8,9,10]. Indicators of psychological distress (PD), including depression and burnout, are also common among dental team members, who work in an environment with high occupational stress and suicide rates [11]. Other occupational health-related problems in dentistry such as ocular- and vision-related occupational health issues, percutaneous injuries, noise, allergies, infections, and mercury exposure are also widely discussed [12,13,14,15,16]. The course “Work Safety and Ergonomics in Dentistry” at the Medical University of Silesia, Poland, is offered during the first year of dental studies (second semester). It comprises 30 h (5 h of lectures, 10 h of seminars, and 15 h of practical exercises) and covers various aspects of ergonomics across different dental fields as it is delivered by faculty members from all major dental departments (Conservative Dentistry with Endodontics, Dental Surgery, Dental Prosthodontics, Orthodontics, Pediatric Dentistry, and Dental Materials Science). Students’ knowledge and practical skills are reinforced and consolidated each subsequent semester during theoretical and practical dental exercises. This study aimed to assess the knowledge and awareness of undergraduate students of ergonomics in dentistry and its practical application. Secondary objectives included investigating the prevalence of musculoskeletal pain (MSP) among dental students, assessing their engagement in physical activities as a preventive measure for MSDs, and evaluating students’ self-reported stress levels during dental procedures. Also, the ubiquity of cigarettes and e-cigarette addiction among future dental practitioners were important features of the study. 

## 2. Materials and Methods

This survey-based exploratory research was conducted between 6 and 27 February 2023 using a cross-sectional approach. The study targeted senior undergraduate students (fourth and fifth year) at the Dental Faculty of the Medical University of Silesia in Katowice, Poland. Participants were recruited via convenience sampling, which was chosen for its efficiency in obtaining a suitable sample relevant to the research project requirements. The recruitment process involved researchers approaching students during scheduled lectures, tutorials, or clinical exercises, and inviting them to participate and complete a questionnaire. Students who were willing to participate were given time to complete and submit the questionnaire by the end of their scheduled university class the following day. Participants were informed about the study’s aims and protocol, and accession was voluntary. Verbal consent was obtained from each subject, and those who declined were excluded. Junior undergraduate students (first, second, and third year) were excluded due to insufficient clinical experience necessary to comprehend the study objectives; no other exclusion criteria were applied. The sample size was estimated using the Raosoft application with a 5% margin of error and 95% confidence interval, resulting in a target of 158 subjects. Confidentiality was maintained throughout the study period. The research adhered to the principles of the Declaration of Helsinki and was approved by the Institutional Review Board of the Medical University of Silesia (PCN/CBN/0052/KB/38/23).

The originally designed and validated online questionnaire consisted of 19 self-administered, closed-ended questions. It was divided into three main sections: students’ general characteristics (seven items), two questions regarding the frequency and severity of musculoskeletal pain, and one question about the severity of self-reported stress during dental procedures; attitudes toward ergonomics as a university course subject (three questions); utility of ergonomics in dental clinical practice (one item); and knowledge of ergonomics (five questions). Students self-reported their level of musculoskeletal pain after practical exercises involving patient treatment using a visual analog scale ranging from 1 (no pain) to 5 (very severe pain). Similarly, the level of students’ stress during dental procedures was reported using a visual analog scale varying from 1 (normal) to 5 (very severe). Attitudes were assessed with three questions offering two possible answers each. A positive attitude was scored as 1, and a negative attitude as 0. The total attitude score ranged from 0 to 3, with scores of 0 indicating a poor attitude toward ergonomics, 1 indicating a fair attitude, and 2 or 3 indicating good to very good attitudes. Knowledge about dental ergonomics was assessed through five questions, each with four possible answers, of which only one was correct. A correct answer was scored as 1, and incorrect answers as 0. The total knowledge score ranged from 0 to 5, with scores of ≤1 indicating poor knowledge, 2–3 indicating fair knowledge, and ≥4 indicating good knowledge. 

The survey tool was pre-tested on a pilot group of 30 dental students using Cohen’s Kappa coefficient, yielding results between 0.8 and 1 for each question. Statistical analysis was conducted using Microsoft Excel version 19.0. Descriptive statistics summarized data in proportions and percentages, while the chi-square test and Mann–Whitney test were used for quantitative analysis. Results with *p*-values < 0.05 were considered statistically significant. The study protocol followed STROBE guidelines for observational studies [17]. 

## 3. Results

### 3.1. Primary Characteristics of the Studied Group

A total of 184 respondents out of 266 students were included, with a response rate of 69.2%. Table 1 displays the characteristics of the students. Over two-thirds of subjects (70.65%) were female. The number of students who were 23 years old or younger (48.3%) was almost the same as in the group of older students (51.7%). The majority of respondents were in the fourth year (57.6%) of the university course. Two-thirds of students (66.8%) declared that they participated in some sort of physical activity in the form of routine fitness maintenance. A total of 87% of physically active respondents exercised at least once a week. More than half of all subjects (62.5%) experienced musculoskeletal pain (MSP) after treating patients during exercises. A total of 85.3% of suffering students had pain episodes several times a month and 1.6% of them described the pain as very severe. The number of students who felt stressed while performing dental procedures (46.7%) and the number of those who did not (53.3%) were comparable. When asked about their level of stress, only one student (0.5%) answered that it was very severe. A minority of students (14.1%) smoked cigarettes or vaped. 

Table 2 presents the distribution of students’ physical activity, musculoskeletal pain (MSP), stress, and smoking or vaping behaviors, stratified by the characteristics outlined in Table 1. Most female and male students engaged in physical activity outside dental clinical practice (69.2% and 61.1%, respectively), with no significant association observed between gender and physical activity. Similarly, among students aged 23 years or younger, and those older than 23 years, a high proportion reported engaging in physical exercise (64% and 69.5%, respectively), with no significant association between age and physical activity detected. Furthermore, no significant associations were identified between physical activity and academic year, MSP after treating patients during exercises, self-reported stress levels in the previously mentioned situations, or smoking habits. Regarding MSP, 66% of female and 53% of male students reported experiencing pain, with no significant gender-related differences observed. Likewise, no significant associations were found between MSP and age, academic year, self-reported stress levels, or smoking. Concerning stress during patient treatment, 51.5% of females and 35% of males reported feeling stressed, with no significant gender-based differences noted. Similarly, no significant associations were found between stress and age, academic year, MSP after treating patients during exercises, smoking, or physical activity outside studying hours. A minority of both female and male students (27.7% and 33.3%, respectively) reported smoking cigarettes or vaping e-cigarettes, with no significant associations detected between smoking behaviors and age, academic year, physical activity, MSP, or stress levels during patient treatment.

### 3.2. Dental Students’ Attitudes toward the Application of Ergonomic Rules in Dentistry 

We conducted an analysis to explore the relationship between responses to each question regarding dental students’ attitudes and their characteristics outlined in Table 1. The findings are summarized in Table 3. The majority of students (81%) expressed their belief that ergonomics is a valuable subject during the university course. However, no significant associations were observed between this belief and gender, age, academic year, physical activity, MSP, self-reported stress level, or smoking/vaping habits. A significant proportion of participants (85.8%) indicated that lecturers should allocate more attention to ergonomics during practical classes. Interestingly, a significant association was detected between this viewpoint and the prevalence of MSP (*p* < 0.05). Specifically, students experiencing MSP were more likely to advocate for increased attention to ergonomics by lecturers during practical classes (*p* = 0.012).

Furthermore, a substantial majority of students (79.3%) reported that they realistically do not follow ergonomic rules in clinical settings. Yet, no significant associations were identified between this perception and gender, age, academic year, physical activity, MSP, self-reported stress, or smoking/vaping habits. Most of the respondents (79.4%) declared that they did not work in an ergonomic position.

Students demonstrated different attitudes toward ergonomics ranging from very good (14.7%), good (61.4%), and fair (20.1%) to poor (3.8%).

**Figure 1:**
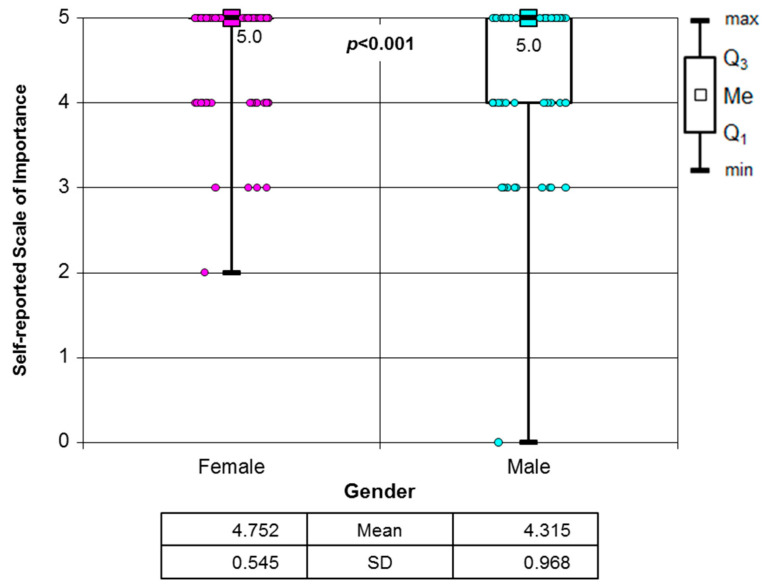
Importance of ergonomic rule application in dentists’ clinical work vs. gender (*p* < 0.001, Mann–Whitney test).

The subjects were also asked how much do they find ergonomics important in dentists’ clinical work (on a five-point scale). Almost three-quarters of students (72.3%) declared that ergonomics is very important. A significant association between gender and this opinion was observed. Women find dental ergonomic principles to be a crucial, integral element of dental practice (*p* < 0.001) (Figure 1).

No other association between this perception and age, academic year, physical activity, MSP, self-reported stress, or cigarettes/vaping has been found. 

### 3.3. Dental Students’ Knowledge Related to Ergonomics

Table 4 displays students’ knowledge of ergonomics addressing dental aspects. The majority (57.1%) of respondents answered incorrectly regarding what is four-handed dentistry. A proportion of students revealed an adequate knowledge of an operator’s correct position during dental procedures (87.5%), how all dental team members should be placed during dental procedures (79.3%), and which domains of the operator’s body are under the biggest burden while performing procedures (87%). Only one-third of participants, however, were competent enough to describe the optimal patient’s position from an ergonomics perspective (37%). In the second question, fourth-year student answers had more correct answers (*p* = 0.032). In the third question, fifth-year students gained better results (*p* = 0.039).

The majority of students demonstrated good (47.9%) and fair (46.7%) levels of knowledge, revealing a significant association between their knowledge and age (0.010), as well as academic year (*p* = 0.008). Reportedly, younger students had inadequate knowledge on the subject of ergonomics. Also, students who had good knowledge of the topic of dental ergonomics demonstrated lower engagement in physical activity (0.046). The relationship between students’ knowledge and gender, MSP, stress, or smoking/vaping did not show statistical significance (Table 5).

## 4. Discussion

The presented research reinforces the existing body of knowledge in the field of dental ergonomics by corroborating previous findings. Both students and dental professionals have predominantly fair to good knowledge regarding ergonomic principles and positive attitudes toward it. However, a high proportion of dental students and practitioners self-report ‘bad’ occupational habits and practices [18,19]. This common recurring pattern indicates that the practical application of ergonomic principles is still inadequate despite declared self-awareness of its importance. Although no novel findings emerged from our study, the results provide robust evidence that emphasizes the continued relevance and importance of ergonomic interventions in dental practice. Life-long learning and iteration of ergonomic rules during years of postgraduate clinical practice are deemed to be key to maintaining good physical and psychological health of future generations of dental practitioners and other members of the dental team.

Based on our presented study, ergonomics in dentistry has been sorely neglected both in universities and postgraduate training curricula. Hence, the vast majority of our respondents highlighted the urge for lecturers to pay more attention to ergonomic principles during clinical dental training procedures; this was especially expressed by those students who already suffer from MSDs. This need has also been concluded by other authors [1]. 

In the presented paper, there was a significant association between students’ core ergonomic knowledge and advancement in dental study. This was in agreement with another study carried out El-sallamy et al. who reported that a significant difference in knowledge was observed between studied subjects with an increase in their academic level [20]. 

There was one appreciable difference between the demographic variables of the participants where females seemed to rank the importance of ergonomics in dental practice higher than men [21]. The feeling of exhaustion and MSP after clinical procedures tend to be gender-related, which is why the result that showed women find ergonomics more significant and useful did not come as a surprise [22]. 

In this study, 33.7% of dental students are not engaged in physical exercise. Physical inactivity among future dentists seems to put them at high risk of WMSDs, especially without the application of ergonomic principles [23,24]. Exercise has a great positive impact on the reduction of MSP. Koneru et al. found a significant decrease in the frequency of MSP among dentists who practiced yoga or were engaged in regular physical exercise compared to those who did not. Additionally, it was found that yoga is the most effective form of physical activity, considering its controlled nature and beneficial effects on reducing stress and mental tension [25]. 

Due to the demanding nature of the dental profession, MSDs commonly experienced by dentists have been extensively examined, showing an approximate prevalence of 78% [26]. These conditions have also emerged as a significant health concern within the Polish healthcare sector [27]. A recent systematic review revealed that while the neck, elbows, and lower back showed similar rates and prevalence of MSDs between dental students and practitioners, the rest of the examined body regions showed higher rates of MSDs among dental students. Possible explanations may be that students do not fully adopt the optimal techniques and ergonomic strategies to carry out dental procedures, leading to greater physical strain [28]. In a study by Sangalli at el., which involved fourth- and fifth-year dental students, MSP was reported by the majority of participants, with a prevalence of 53.9% after commencement of clinical procedures [29].

Stress, a dynamic adaptive relationship between the abilities of an individual and the requirements of a situation, is an inseparable part of our lives. If environmental stressors remain for too long, they can lead to the impairment of effective thinking and learning, and, in the long term, to serious mental and physical disorders [30]. In a study performed by Mocny-Pachońska et al. at the Medical University of Silesia, Poland, the prevalence of highly perceived stress varied from 4.3% to 47.3% depending on the type of dental procedure performed (conservative and endodontic treatment, respectively) and decreased with the year of advancement in the university course [31].

In a study by Rodakowska et al., 28% of dental students from Bialystok University, Poland, admitted to tobacco smoking or vaping in the last 30 days, which closely aligns with the findings presented in our study [32]. Regarding the fact that dental students are constantly educated on the adverse effects of smoking on oral and general health, the overall prevalence of nicotine use among students is considered high. 

The practical implication of the presented study should include the enhancement of training programs in dental ergonomics in both pre- and postgraduate education. Dentists should be encouraged to implement and abide by ergonomic standards, health and safety regulations, and policies that promote work–life balance in dental practice environments for the sake of their future physical and mental health. 

Future research should consider conducting longitudinal studies to examine the long-term consequences of dentists’ lack of compliance with ergonomic principles. Combining perspectives from different disciplines, such as dentistry, dental technology, ergonomics, and psychology, could provide a more holistic understanding of the studied issues and might uncover relationships and insights that single-discipline studies might miss. By addressing these practical implications and exploring the suggested directions for future research, we can enhance application of the study’s findings, contributing to an increase in dentists’ awareness about the consequences of the lack of application of ergonomic principles in everyday dental practice. 

### Limitations of the Study

The students may have responded as anticipated and not according to their actual practice patterns (social desirability bias and cognitive response bias). The junior students may have found it challenging to answer some of the questions due to their limited clinical experience. Also, closed-ended, single-answer questionnaires often provide limited opportunity for participants to provide more nuanced responses. Addressing these limitations should involve qualitative research methods (interviews and observations), which would mitigate biases and maximize the quality of data obtained. The sample size, although adequate considering statistical impact, may require further multi-center validation using larger study groups, including numerous dental schools and different student characteristics. 

## 5. Conclusions

Overall, we conclude that dental students have predominantly fair to good knowledge and a positive attitude toward ergonomics, which is incoherent with its lack of application during routine procedures in clinical settings. Acquiring desirable ergonomic habits requires its proper early introduction, constant supervision, and enhanced implementation, consistently reinforced by dental university teachers. Hence, there is an urgent need to incorporate principles of ergonomics into all preclinical dental curricula in the context of practical, real clinical workload. Also, regular training programs throughout the clinical years of university courses to improve knowledge and attitude, and solidify good practices are crucial. 

## Figures and Tables

**Table 1:** Characteristics of studied group.

Characteristics		n (%)
Gender	Female Male	130 (70.7) 54 (29.3)
Age (years)	≤23 >23	89 (48.3) 95 (51.7)
Academic year	4th 5th	106 (57.6) 78 (42.4)
Physical activity outside studying hours	Yes No	123 (66.8) 61 (33.2)
Musculoskeletal pain after treating patients during practical exercises	Yes No	115 (62.5) 69 (37.5)
Feeling of stress while performing dental procedures	Yes No	86 (46.7) 98 (53.3)
Cigarette and e-cigarette smoking	Yes No	26 (14.1) 158 (85.9)

**Table 2:** Students’ physical activity, musculoskeletal pain, stress, and smoking or vaping according to their characteristics.

Students’ Characteristics	Physical Activity		*Χ*^2^ (*p*)	Musculoskeletal Pain		*Χ*^2^ (*p*)	Stress		*Χ*^2^ (*p*)	Cigarettes/ E-Cigarettes		*Χ*^2^ (*p*)
	n (%)			n (%)			n (%)			n (%)		
	Yes	No		Yes	No		Yes	No		Yes	No	
			0.798			2.020			3.468			0.345
Gender			0.372			0.155			0.063			0.557
Female	90 (48.9)	40 (21.7)		86 (46.7)	44 (23.9)		67 (36.4)	63 (34.2)		36 (19.6)	94 (51.1)	
Male	33 (17.9)	21 (11.4)		29 (15.8)	25 (13.6)		19 (10.3)	35 (19.0)		18 (9.7)	36 (19.6)	
			0.391			0.001			0.105			0.595
Age			0.532			0.970			0.745			0.441
≤23	57 (31.0)	32 (17.4)		56 (30.4)	33 (17.9)		40 (21.7)	49 (26.6)		29 (15.8)	60 (32.6)	
>23	66 (35.9)	29 (15.8)		59 (32.1)	36 (19.6)		46 (25.0)	49 (26.6)		25 (13.6)	70 (38.0)	
			1.331			0.291			0.000			0.614
Academic year			0.249			0.590			0.990			0.433
fourth	75 (40.8)	31 (16.8)		64 (34.8)	42 (22.8)		50 (27.2)	56 (30.4)		34 (18.5)	72 (39.1)	
fifth	48 (26.1)	30 (16.3)		51 (27.7)	27 (14.7)		36 (19.6)	42 (22.8)		20 (10.9)	58 (42.4)	
						1.192			0.000			2.500
Physical activity						0.275			0.997			0.114
Yes				73 (39.7)	50 (27.2)		57 (31.0)	66 (35.9)		31 (16.8)	92 (50.0)	
No				42 (22.8)	19 (10.3)		29 (15.8)	32 (17.4)		23 (12.5)	38 (20.7)	
Musculoskeletal Pain (MSP)			1.192						0.006			0.007
			0.275						0.939			0.933
Yes	73 (39.7)	42 (22.8)					54 (29.3)	61 (33.2)		34 (18.5)	81 (44.0)	
No	50 (27.2)	19 (10.3)					32 (17.4)	37 (20.1)		20 (10.9)	49 (26.6)	
			0.000			0.006						0.790
Stress			0.997			0.939						0.374
Yes	57 (31.0)	29 (15.8)		54 (29.3)	32 (17.4)					22 (12.0)	64 (34.8)	
No	66 (35.9)	32 (17.4)		61 (33.2)	37 (20.1)					32 (17.4)	66 (53.3)	
Cigarettes/ e-cigarettes			2.500			0.007			0.790			
			0.114			0.933			0.374			
Yes	31 (16.8)	23 (12.5)		34 (18.5)	20 (10.9)		22 (12.0)	32 (17.4)				
No	92 (50.0)	38 (20.7)		81 (44.0)	49 (26.6)		64 (34.8)	66 (35.9)				

**Table 3:** Attitudes and practices of dental students and their characteristics. The bold and italic data means *p*-value < 0.05.

Students’ Characteristics	Question A1 *		*Χ*^2^ (*p*)	Question A2 **		*Χ*^2^ (*p*)	Question A3 ***		*Χ*^2^ (*p*)
	n (%)			n (%)			n (%)		
	Yes 149 (81)	No 35 (19)		Yes 158 (85.8)	No 26 (14.1)		Yes 38 (20.7)	No 146 (79.3)	
			1.773			3.235			3.024
Gender			0.183			0.072			0.082
Female	109 (59.2)	21 (11.4)		116 (63.0)	14 (7.6)		22 (12)	108 (58.7)	
Male	40 (21.7)	14 (7.6)		42 (22.8)	12 (6.5)		16 (8.7)	38 (20.7)	
			1.801			2.98			0.166
Age			0.18			0.084			0.683
≤23	68 (37.0)	21 (11.4)		81 (44)	8 (4.3)		20 (10.9)	69 (37.5)	
>23	81 (44.0)	14 (7.6)		77 (41.8)	18 (9.8)		18 (9.8)	77 (41.8)	
			0.789			0.05			0.924
Academic year			0.374			0.823			0.336
fourth	83 (45.1)	23 (12.5)		90 (48.9)	16 (8.7)		25 (13.6)	81 (44)	
fifth	66 (35.9)	12 (6.5)		68 (37)	10 (42.4)		13 (7.1)	65 (35.3)	
			0.128			0.1057			2.513
Physical activity			0.72			0.692			0.113
Yes	101 (54.9)	22 (12.0)		107 (58.2)	16 (8.7)		30 (16.3)	93 (50.5)	
No	48 (26.1)	13 (7.1)		51 (27.7)	10 (5.4)		8 (4.3)	53 (28.8)	
			0.849			***6.318***			0.221
MSP			0.357			***0.012***			0.638
Yes	96 (52.2)	19 (10.3)		105 (57.1)	10 (5.4)		22 (12)	93 (50.5)	
No	53 (28.8)	16 (8.7)		53 (28.8)	16 (8.7)		16 (8.7)	53 (28.8)	
			0.105			0.022			3.687
Stress			0.746			0.883			0.055
Yes	71 (38.6)	15 (8.2)		74 (40.2)	12 (6.5)		12 (6.5)	74 (40.2)	
No	78 (42.4)	20 (53.3)		84 (45.7)	14 (7.6)		26 (14.1)	72 (39.1)	
			0.009			1.779			1.125
Cigarettes			0.925			0.182			0.289
Yes	44 (23.9)	10 (5.4)		43 (23.4)	11 (6.0)		8 (4.3)	46 (25)	
No	105 (57.1)	25 (13.6)		115 (62.5)	15 (8.2)		30 (16.3)	100 (54.3)	

* Question A1 Do you think that ergonomics is a useful subject during the university course? ** Question A2 Do you think that the lecturers should pay more attention to the ergonomic position of students during clinical procedures? *** Question A3 Do you think that you work ergonomically during clinical procedures?

**Table 4:** Students’ general knowledge related to dental ergonomics and their characteristics. The bold and italic data means *p*-value < 0.05.

Students’ Characteristics	Question K1 *		*Χ*^2^ (*p*)	Question K2 **		*Χ*^2^ (*p*)	Question K3 ***		*Χ*^2^ (*p*)	Question K4 ****		*Χ*^2^ (*p*)	Question K5 *****		*X*^2^ (*p*)
	n (%)			n (%)			n (%)			n (%)			n (%)		
	Correct 79 (42.9)	Incorrect 105 (57.1)		Correct 161 (87.5)	Incorrect 23 (12.5)		Correct 146 (79.3)	Incorrect 38 (20.7)		Correct 160 (87.0)	Incorrect 24 (13.0)		Correct 68 (37.0)	Incorrect 116 (63.0)	
			1.453			0.374			0.291			0.551			0.239
Gender			0.228			0.541			0.59			0.458			0.625
Female	60 (32.7)	70 (38)		112 (60.9)	18 (9.8)		105 (57.1)	25 (13.6)		111 (60.3)	19 (10.3)		50 (27.2)	80 (43.5)	
Male	19 (10.3)	35 (19)		49 (26.6)	5 (2.7)		41 (22.3)	13 (7.1)		49 (26.7)	5 (2.7)		18 (9.8)	36 (19.6)	
			0.26			1.371			1.292			0.686			0.014
Age			0.61			0.242			0.256			0.407			0.905
≤23	36 (19.6)	53 (28.8)		81 (44.0)	8 (4.3)		67 (36.4)	22 (12.0)		75 (40.8)	14 (7.6)		33 (17.9)	56 (30.4)	
>23	43 (23.4)	52 (23.2)		80 (43.5)	15 (8.2)		79 (42.9)	16 (8.7)		85 (46.2)	10 (5.4)		35 (19.0)	60 (32.6)	
			0.367			4.591			4.272			0.55			2.087
Academic year			0.544			***0.032***			***0.039***			0.458			0.149
fourth	43 (23.4)	63 (34.2)		98 (53.3)	8 (4.3)		78 (42.4)	28 (15.2)		90 (48.9)	16 (8.7)		34 (18.5)	72 (39.1)	
fifth	36 (19.6)	42 (22.8)		63 (34.2)	15 (8.2)		68 (37.0)	10 (5.4)		70 (38.0)	8 (4.3)		34 (18.5)	44 (23.9)	
			1.096			1.012			0.001			0.045			0.096
Physical activity			0.295			0.314			0.97			0.832			0.756
Yes	49 (26.6)	74 (40.2)		105 (57.1)	18 (9.8)		97 (52.7)	26 (14.1)		106 (57.6)	17 (9.2)		44 (23.9)	79 (42.9)	
No	30 (16.4)	31 (16.8)		56 (30.4)	5 (2.7)		49 (26.6)	12 (6.5)		54 (29.3)	7 (3.8)		24 (13.0)	37 (20.2)	
			0.427			0.268			2.556			1.278			0
MSP			0.513			0.604			0.11			0.258			1
Yes	52 (28.3)	63 (34.2)		99 (53.8)	16 (8.7)		96 (52.7)	19 (10.3)		97 (52.7)	18 (9.8)		43 (23.4)	72 (39.1)	
No	27 (14.7)	42 (22.8)		62 (33.7)	7 (3.8)		50 (27.2)	19 (10.3)		63 (34.2)	6 (3.3)		25 (13.6)	44 (23.9)	
			0.03			0.012			0.403			0.099			0.692
Stress			0.863			0.911			0.526			0.753			0.406
Yes	38 (20.7)	48 (26)		76 (29.3)	10 (17.4)		66 (35.9)	20 (10.9)		76 (41.3)	10 (5.4)		35 (19.0)	51 (27.8)	
No	41 (22.3)	57 (31)		85 (33.2)	13 (20.1)		80 (43.5)	18 (9.8)		84 (45.7)	14 (7.6)		33 (17.9)	65 (35.4)	
Cigarettes/ e-cigarettes			0.011			0.374			0.019			2.761			1.413
			0.918			0.541			0.889			0.097			0.235
Yes	23 (12.5)	31 (16.8)		49 (26.6)	5 (2.7)		42 (22.8)	12 (6.5)		43 (23.4)	11 (6.0)		24 (13.0)	30 (16.4)	
No	56 (30.4)	74 (40.2)		112 (60.9)	18 (9.8)		104 (56.5)	26 (14.1)		117 (63.6)	13 (7.0)		44 (23.9)	86 (46.7)	

* Question K1 What is four-handed dentistry? ** Question K2 What should the correct position of an operator during a dental procedure look like? *** Question K3 How should the dental team members be placed while performing procedures? **** Question K4 Which areas of the dentist’s body are under the biggest burden while performing clinical procedures? ***** Question K5 How should the patient be positioned during dental procedures?

**Table 5:** Students’ characteristics reflecting their knowledge on ergonomic principles. The bold and italic data means *p*-value < 0.05.

Students’ Characteristics	Knowledge			*Χ*^2^ (*p*)
	n (%)			
	Good 88 (47.9)	Fair 86 (46.7)	Poor 10 (5.4)	
				2.34
Gender				0.310
Female	62 (33.7)	63 (34.2)	5 (2.7)	
Male	26 (14.1)	23 (12.5)	5 (2.7)	
				9.236
Age				***0.010***
≤23	45 (24.5)	35 (19.0)	9 (4.9)	
>23	43 (23.4)	51 (27.7)	1 (0.5)	
				9.644
Academic year				***0.008***
fourth	53 (28.8)	43 (23.4)	10 (5.4)	
fifth	35 (19.0)	43 (23.4)	0 (0.00)	
				6.145
Physical activity				***0.046***
Yes	51 (27.7)	65 (35.3)	7 (3.8)	
No	37 (20.1)	21 (11.4)	3 (1.6)	
				2.135
MSP				0.344
Yes	59 (32.1)	49 (26.6)	7 (3.8)	
No	29 (15.8)	37 (20.1)	3 (1.6)	
				1.905
Stress				0.386
Yes	44 (23.9)	36 (19.6)	6 (3.3)	
No	44 (23.9)	50 (27.2)	4 (2.2)	
Cigarettes/e-cigarettes				5.195
Yes	30 (16.3)	67 (36.4)	5 (2.7)	0.074
No	58 (31.5)	19 (10.3)	5 (2.7)	

## Data Availability

Data are contained within the article.
